# Informing Active Play and Screen Time Behaviour Change Interventions for Low Socioeconomic Position Mothers of Young Children: What Do Mothers Want?

**DOI:** 10.1155/2016/2139782

**Published:** 2016-12-08

**Authors:** Katherine L. Downing, Keren Best, Karen J. Campbell, Kylie D. Hesketh

**Affiliations:** Institute for Physical Activity and Nutrition (IPAN), School of Exercise and Nutrition Sciences, Deakin University, Geelong, VIC, Australia

## Abstract

*Introduction*. This study investigated views of mothers from disadvantaged urban and regional areas (i.e., beyond major capital cities) as potential end users of child active play and screen time behaviour change interventions, with a focus on text messaging and web-based delivery platforms.* Methods*. Thirty-two mothers (22 urban; 10 regional) were interviewed. Purpose-designed questions covered topics regarding mothers' preferences for accessing and receiving information related to parenting and child active play and screen time. Data from transcribed interviews were analysed to identify responses and key themes.* Results*. Mothers reported frequently accessing parenting- and child-related information online. Regional mothers reported seeking information by talking with other people less frequently than urban mothers and seemed to have a stronger preference for receiving information online. There were few differences between responses from low and high educated mothers. The majority of mothers reported that they would be happy to receive text messages containing information about active play and screen time and that they would find a dedicated website with this information useful.* Conclusions*. Mothers in this study held favourable views on the potential of receiving information via new communication technologies. Future interventions targeting socioeconomically disadvantaged mothers may benefit from delivering intervention messages via these technologies.

## 1. Introduction

Good intervention development practice involves inclusion of key stakeholders and/or end users in the intervention development phase [1, 2]. This has been shown to lead to greater acceptability and engagement with interventions and improved efficacy. Involvement of end users in intervention development may be particularly pertinent in groups who are difficult to engage. Those of low socioeconomic position (SEP) are known to be difficult to engage in behaviour change interventions and are consistently underrepresented in published behaviour change research [3–5], despite being the group at highest need. This imbalance needs to be redressed to ensure that behaviour change interventions do not inadvertently work to increase the health disparities in our society. New communication technologies may hold promise in this regard.

The emergence of new communication technologies has seen a rapid change in the delivery of behaviour change interventions over the past decade. Much of this change involves a move from face-to-face or voice call (telephone) intervention delivery to delivery by remote communication technologies such as websites, apps, and short messaging services (text messages delivered to mobile phones). Such evolution and the greater user flexibility offered by new communication technology delivery may be of benefit to difficult to reach groups, such as those of low SEP. Specifically, such delivery modes allow individuals to access interventions at a time and place convenient to them, to work through interventions at their own pace, allow for more visual and less didactic learning, and allow for a broader range of learning and feedback [6].

Those with additional constraints that may further limit their ability to engage with traditionally delivered interventions, for example, parents with young children, may be particularly benefited by the flexibility afforded by these technologies. Intervention delivery via text messages has not been widely used in early childhood studies to date [7]. However, a recent pilot intervention delivered largely via text messages, focusing on improving parental knowledge regarding nutrition and physical activity for overweight and obese preschoolers, was shown to be effective, feasible, and acceptable [8]. Given our limited insights regarding our ability to engage with these groups, it is important to seek better describing and understanding these end users' preferences for how they best like to learn and receive behaviour change information.

Mothers in the Australian context provide the majority of child care [9] and as such may have the largest influence on a wide range of child behaviours. For this reason, mothers are commonly targeted in child behaviour change interventions. Engagement in playtime and active play from a young age has important health and developmental benefits [10, 11]. Conversely, engagement in high levels of screen time is associated with a number of detrimental health and developmental outcomes in early childhood [12]. Therefore, feasible and acceptable strategies to promote increased active play and decreased screen time at this young age are necessary. However, to date behaviour change interventions in this group have been scarce and have primarily relied on traditional delivery modes.

This study focused on mothers with young children (1–3 years of age) from low SEP urban and regional areas (i.e., areas beyond the major capital cities) as potential end users of early childhood behaviour change interventions. It aimed to gauge end user views on preferred methods and forms of receiving active play and screen time behaviour change information, with a particular focus on text messaging and web-based delivery platforms.

## 2. Methods

This study involved face-to-face interviews with mothers from both urban and regional low SEP areas in Victoria, Australia. The interviews were designed to collect both quantitative and qualitative data on mothers' preferences for accessing and receiving information related to their child's active play and screen time. Data were collected from November to December 2013. Ethics approval was received from the Faculty of Health Human Ethics Advisory Group at Deakin University.

### 2.1. Participant Recruitment

A local government area (LGA) from an urban area and a regional area representing the lowest decile for socioeconomic disadvantage (i.e., the most disadvantaged, based on the Australian Bureau of Statistics Socioeconomic Indexes for Areas (SEIFA) [13]) were purposively selected based on their proximity to Deakin University, Burwood Campus. Maternal and Child Health Centres (MCHCs) and playgroups (informal gatherings for parents/caregivers and their 0- to 5-year-old children prior to the commencement of school) within these areas were contacted by the researchers and asked to distribute flyers outlining and inviting study participation to mothers. In response to initial low recruitment uptakes the study broadened its advertising via the use of community noticeboards and advertisements in the local paper of the regional area. Interested mothers were asked to contact the research team who assessed eligibility (i.e., having a child aged 1 to 3 years and being able to speak, read, and write fluent English) and arranged a convenient time and place to conduct the face-to-face interview. Recruitment continued until data saturation was reached.

### 2.2. Procedure

At the interview, mothers completed a 2-page survey reporting their own and their child's basic demographic information (e.g., their own education level, employment status, and marital status and their child's age and sex) and household characteristics including technology in the home (e.g., computer, internet access), the device they used most frequently to access the internet, and the number of hours per week spent browsing the internet for general information. Structured face-to-face interviews involving both closed- and open-ended questions were then conducted with the mothers by a single facilitator. The interview questions were purpose-designed to collect both quantitative and qualitative data regarding mothers' preferences for finding and receiving information about parenting or young children, with a particular focus on active play and screen time. Examples of questions asked included the following: “When you want to know something about parenting or young children where do you go to get information?”; “How do you best like to learn about things related to your child and parenting?”; and “Have you ever looked for information about young children's play?” For open-ended questions, mothers were prompted until no new avenues were volunteered. Specific questions were also asked regarding what sort of information mothers would find useful if they were to be involved in a program where information on children's play and screen time was provided to them, how they would feel about receiving the information via text messages, emails, or websites, and how often they would prefer to receive the information via these channels. Mothers were also asked about their opinion about specific features of websites, namely, the inclusion of pictures, informative videos, and written information.

The interviews included a participatory activity, whereby mothers were asked to look at and browse two different child/parenting websites on an iPad and provide feedback on what they liked and did not like. The order in which the websites were shown to mothers was alternated randomly to ensure participants were not always comparing one website to the other. The facilitator inconspicuously took note of where mothers browsed (i.e., the topics/sections of the website they clicked on). Finally, mothers were asked a broad question regarding their suggestions for researchers designing a program and resources for parents with information about children's play and screen time.

Interviews lasted from 20 to 40 minutes. The interviews were recorded using a small electronic recording device. A second facilitator was present at all interviews to assist with logistics including childcare. Participants were given a $20 gift voucher at the completion of the interview in recognition of their time.

### 2.3. Data Management and Analysis

Demographic survey data were analysed in Stata 14.0 (StataCorp, Texas, USA). Descriptive statistics were used to describe the maternal, child, and household characteristics. *t*-tests and chi-squared tests were used to determine differences in characteristics between urban and regional participants. Recorded interviews were transcribed verbatim by an external transcription company. Analyses were undertaken in the qualitative software package NVivo (QSR International, 2002). Participants' responses to questions were coded by a single researcher (KLD) to identify responses to closed-ended questions and to identify key themes in open-ended questions. All codes were confirmed by the interview facilitator (KB).

## 3. Results

In total, 32 mothers were recruited: 22 from the urban area and 10 from the regional area. Maternal, child, and household characteristics are presented in Table 1. Mean (SD) maternal age was 33.4 (6.1) years. Just under half of all mothers had a university degree. Urban mothers had higher levels of employment with 59.1% working either full or part-time for an average of 14.5 hours per week compared with 30% of regional mothers working part-time (none full time) for an average of 8.3 hours per week; however, this was not significantly different. The only statistically significant difference between urban and regional mothers was marital status: 95.5% of urban mothers were cohabitating with a partner compared to 40.0% of regional mothers (*p* = 0.01). The mean (SD) child age was 2.5 (0.9) years. Mothers reported that their child lived with them 100% of the time. All mothers had access to a mobile phone, with all but one urban and one regional mother reporting internet access on their mobile phones. Mothers most often accessed the internet via their mobile phone and spent around seven hours per week browsing the internet for general information. Given that almost half of all mothers recruited had a university degree (i.e., may not necessarily be considered to be low SEP), results are presented by urban/regional classification and by education level. “Low educated” was defined as having less than a university degree while “high educated” was defined as having a university degree or higher qualification.

### 3.1. Accessing Information

A summary of mothers' responses to questions regarding accessing information is presented in Table 2. Both urban and regional mothers reported that they used a range of different sources to access information about parenting and their child. The most commonly reported sources were the internet (including Google search, specific websites, Facebook forums, and email newsletters; 84%), personal advice from friends, family, or other parents (59%), or health professionals (most commonly a maternal and child health nurse; 50%). Urban and high educated mothers reported using the internet more frequently than regional and low educated mothers. In addition, high educated mothers seemed to report reading books/magazines more frequently than low educated mothers. Mothers frequently reported using different sources for different types of information sought and personal advice was sought both in person and online.

*I have an online Mum's group on Facebook, so a lot of people post questions on there and I can ask advice there if I want first-hand info, otherwise Google if I'm trying to find say a serving size of something or how many hours a baby might normally sleep.* (Regional, low educated mother)

*I try Google first. That's the first thing I would do and then, maybe… we go to a play group on Friday mornings, so yeah, we kind of bounce around the ideas between us mothers.* (Urban, high educated mother)

*Depends what it is but either the internet, maybe speaking to another parent or my parents. If he's due for a maternal health visit I'll ask them.* (Regional, high educated mother)

Consistent with the preferred ways of accessing information, talking with lay people (e.g., friends, family; 75%) and the internet (44%) were commonly reported as ways in which mothers best like to learn. Additionally, almost half (47%) of all mothers reported a preference for learning by reading books, magazines, or other written materials. For the most part, urban and regional and low and high educated mothers reported the same preferences for learning; however, regional and high educated mothers less frequently reported a preference for talking with people than urban and low educated mothers. Many mothers discussed the need to get information from a variety of sources as children and families are all different and strategies that worked for one person may not work for others.

*I like to get as many information as you can and then just form your own idea from them rather than just trust one thing… trust just only somebody to think and you don't do your own research. I don't do that. I do my own research. I still talk with people and from them… from their ideas I pick up the ones that I think is right… is suitable for our family… because family they're different.* (Urban, high educated mother)

*Mostly, I like first-hand advice because the nurses, when we go for her check-up, they have good advice but, everyone has different ideas, so I take first-hand advice and take it or leave it, because it might not work for us.* (Regional, low educated mother)

### 3.2. Children's Play and Screen Time Information

The majority of mothers reported that they had previously looked for information on children's play and screen time; however, a much higher proportion of high educated mothers reported that they had previously looked for information on children's play than low educated mothers (93% versus 41%). The most commonly reported searches were for developmental milestones or age-appropriate activities (*n* = 11), recommendations for screen time (*n* = 10), and activities/craft ideas (*n* = 8). Mothers mostly searched for this information online or in books. Some mothers reported that they often looked for new play or activity ideas due to limited space at home or poor accessibility to indoor play areas.

*Yeah, there's a couple of sites that I've looked at because sometimes… well, especially in winter when it's raining and we've got a fairly small house and you feel a bit… you can only go to play centres so often and you want to make it… What else can I do with the kids that's outside?* (Urban, high educated mother)

*I think for me living where I'm living, it's different to the city because in the city there's so many places you can access to take your kids whereas down here it's like… what do I do, there's not a lot. Like up there they have play centres and stuff they don't have down here, so you're limited to perhaps finding a park with the weather permitting or… that's why I did go online to try and find activities to do at home.* (Regional, high educated mother)

### 3.3. Program Preferences

Mothers were asked how they would prefer to receive information about children's play and screen time if they were to be involved in a program that provided such information. A summary of urban and regional and low and high educated mothers' responses is presented in Table 3. Interestingly, one of the most commonly reported sources for urban mothers (50%) was paper-based, written information. Half of the urban mothers also reported that they would be happy to receive this information via email. Regional mothers most commonly reported that they would prefer to receive program information online or via email, with only 30% of mothers mentioning paper-based information as a preferred form. One-third of mothers with high education level mentioned online delivery as a preferred form, compared to 18% of mothers with low education. When prompted about whether they would be happy to receive text messages containing this type of information, the majority of mothers (>70%) agreed, with little difference between urban/regional or low/high educated mothers. Mothers mostly reported that they would like to receive information text messages once per week. The vast majority of mothers (91%) agreed that having links in the text messages to reputable websites would be useful. When asked how they would feel about having a dedicated website to go to with information about children's play, the majority of mothers (91%) responded that would like and use it.

*Yeah, that'll be fantastic because a lot of the other websites have food, they have problems with behaviour, they have baby names, they have a huge range of things, so one just for play would be fantastic.* (Urban, high educated mother)

Responses to how often mothers would visit such a website were varied; however, the most commonly reported frequency for mothers was once per week. Mothers were overwhelmingly positive about receiving email reminders to visit such a website and reported that they would be happy to receive them once per week to once per fortnight.

### 3.4. Websites

Prior to being shown the two parenting/child websites, mothers were asked to think about websites in general and to comment on what makes a website one that they enjoy visiting. One of the most frequently reported factors for both urban and regional mothers was that the website should be easy to navigate and find information, for example, having a search tool.

*Something with a search box at the top or the bottom somewhere so, if I can't see exactly where I'm looking for it I can just search a keyword because sometimes I can't always find what I'm looking for.* (Regional, low educated mother)

*One where you can find the information easily and that has a search, like a search tool so that you can find what you're looking for quite quickly.* (Urban, high educated mother)

Another common theme was that mothers liked websites that were well-designed and simple, without too much clutter or information.

*The design of the website is attractive, not too busy. There's too many websites have so much going on that's it's confusing to the eye. It has to be user friendly so when you want to get from one section to the next it's easy and it's not complicated to work out what to do or where to get to or how to retrieve the information you want to retrieve and just not too much information… It depends on what the topic is but I think some people can just dismiss if there's too much going.* (Regional, high educated mother)

When considering their preferences for websites, some mothers commented that they preferred to know that the information came from a credible source or was evidence-based, while some preferred to read advice from other mothers or parents who had had similar experiences.

*Some professional… like very professionals like maybe a professor who's been doing this area on this age group for lots of time. Maybe they have a few books published or… yeah, just the professional ones. If it's just general information you can get it anywhere. Even mothers are… “I have four kids. I have heaps of experience. I can give you heaps of information.” But that's different. Somebody who's studying this, they can be different.* (Urban, high educated mother)

*I like one that has a lot of reviews, like if there's a question and then like a lot of other mothers have put down yep, their story similar to that, so then you're like not feeling alone if your child does that.* (Urban, low educated mother)

Finally, some low educated mothers said that websites should be mobile-friendly, that is, easily accessed and readable on a smartphone.

*That can be used on the mobile because I basically use everything on my mobile.* (Urban, low educated mother)

*Normally like something that's mobile friendly as well. If you're out and you've got time to fill in you can have a look on it, because a lot of ones aren't.* (Regional, low educated mother)

When mothers were shown specific websites, the most common themes that emerged in terms of what they liked were having information categorised by child age, clear and simple layout, and being easy to navigate. Mothers seemed to prefer websites that were more colourful and “eye-catching,” with pictures and videos rather than large chunks of text.

*It's got more colours in it. Very user friendly again, so even people with basic computer skills I think would be able to find their way through the site with no problems and I liked the fact that once you did click on a topic it had… the separated topics were more obvious so it wasn't too much information going through the head. It was easier to find what you wanted within an age group. You could easily see it separated and it was so user friendly to just quickly press the topic and it would come up and it seemed to have a wider range of information available.* (Regional, high educated mother)

*I really liked this website actually. It was very visual. There was pictures, and probably easy to navigate compared to the other one, because it's just everything is there, the little pictures, it had a lot more gadgets, so I'm guessing a lot more information… But it wasn't information overload, it was still kind of broken down, but you just had to go in and find it.* (Urban, high educated mother)

Mothers also commented that they liked links to other websites, as it made the information seem more credible and reputable.

*I like that it gives you links to other websites and that. That's probably a good thing because you know that it's a reputable thing coming from that.* (Regional, low educated mother)

As would be expected, mothers tended to click on sections of the websites and topics that were relevant to the ages of their own children. They also tended to be more interested in specific child behaviours or problems (such as toilet training, separation anxiety, asthma, and allergies) rather than broader topics such as play and learning. Another popular area was recipes and feeding; mothers seemed particularly interested in healthy snack ideas for their children.

When asked specific questions about whether they liked pictures, informative videos, and written information on websites, mothers mostly commented that they liked all three (see Table 4). With regard to written information, in particular, some mothers had certain conditions around the information being easy to read (e.g., dot points or small paragraphs) and accompanied with pictures and/or videos, suggesting that a combination of all three may be preferable for mothers.

*I think it wouldn't be good if it was just videos, or if it was just words, or just information. I think it's good to have both. It kind of keeps you interested and kind of makes you eager to see what it is.* (Urban, high educated mother)

Mothers with high education seemed to be more interested in pictures and informative videos than mothers with low education (93% compared to 82% and 80% compared to 59%, resp.). This was further demonstrated by more than a quarter of mothers with high education (compared to only 12% of mothers with low education) stipulating that they liked written information on websites as long as it was accompanied with pictures and/or videos.

### 3.5. Suggestions from Mothers

Mothers were asked a general question about what they would suggest researchers who were designing a program and resources for parents with information about children's play and screen time should include. One of the major themes for regional mothers (mentioned by 40%) was to have the information accessible online, while only 14% of urban mothers mentioned online accessibility.

*I like the regular updates via the website… Providing resources that people could maybe copy or use in a real practical sense with their children. Links to books and activities and those kinds of things that people could really use would be good. So just to make it really useable and accessible so you can find things and actually use them with your children day to day… If a website's there and it's easier to provide links to information that already exists and then perhaps have a summary of what information other websites are providing and then fill the gaps with what's missing that could be helpful too.* (Regional, high educated mother)

Many mother also discussed the need to provide age-specific information.

*So, have a website. Have it where you'd have tips about both of those things for each age group rather than having it being really general.* (Urban, low educated mother)

*Probably have suggestions of games and activities for the different age and development stages.* (Regional, low educated mother)

One of the other most commonly mentioned themes for both urban and regional mothers was to ensure that all of the information presented to parents is credible and backed up with reputable websites.

*Just it's just reliable… you know, it's proven, rather than opinion… when it comes to kids, everyone has their own opinion, I think.* (Regional, low educated mother)

*And really again that the science behind it, about why you know it's much better for kids to go outside, or why it's much better for them to use their imagination, that sort of stuff.* (Urban, low educated mother)

Finally, a number of mothers mentioned the need to ensure that any information given to parents is realistic and nonjudgemental and takes into consideration that every situation is different.

*Also being I guess… showing some empathy towards as a parent, you know, we all kind of turn towards the TV sometimes, so just that understanding, because I think that straightaway… yeah, if I'm reading something and people are kind of going, “Yeah, it's hard,” then I'm like, “OK, you're with me, you're on my wavelength, so I'm willing to hear what you've got to say.”* (Urban, low educated mother)

*Just to make it positive. I think there's a lot of things around about people saying, you know, “Don't let kids under two watch TV”, and don't let them do this and don't let them do… and it's like well… I think everything serves a purpose and I don't think it… lots of things when they're said like that make you… instead of making you think, “Oh, I could do more of this”, it makes you feel bad about it, and the reality is sometimes them sitting down and playing with an iPad for half an hour is better than me screaming at them… because they're being naughty or because they're doing whatever, so sometimes yes, it might not be the ideal situation but how much of our life really is the ideal situation.* (Urban, high educated mother)

## 4. Discussion

This study aimed to gauge both rural and urban, socioeconomically disadvantaged mothers' views on early childhood behaviour change interventions, with particular focus on preferred methods of receiving child active play and screen time information. Findings from this study may be used to inform the development of remotely delivered behavioural interventions for parents of young children, particularly those that can be difficult to reach (i.e., from socioeconomically disadvantaged and/or regional areas). Importantly, the delivery of programs via text messages and/or online was broadly acceptable for low and high educated, urban, and regional mothers. While few programs to date have been delivered to this population via new technology communication [7], this may be a delivery mode for future programs to consider, particularly for low SEP mothers who are typically difficult to reach.

While responses regarding preferred sources of information about parenting and children were mostly similar for urban and regional mothers, it is interesting to note that regional mothers reported talking with other people for parenting and child information far less frequently than urban mothers. It may be that mothers in regional areas are more isolated and have less opportunity to meet and talk with peers and hence turn to other sources of information such as books, the internet, or health professionals more commonly than their urban counterparts. This regional preference for internet resources was confirmed in responses to a broader question about what mothers would suggest researchers should do if designing a program for parents, where almost half of the regional mothers commented that the information should be accessible online (compared to 14% of urban mothers). Hence, new technology communication modes may be particularly appealing to mothers residing in regional areas.

Many mothers reported that they frequently used multiple sources to access information about their child, often weighing up advice from health professionals or online resources with personal advice from other parents. The idea that children and families are all different and that when it comes to parenting, strategies that worked for one parent may not necessarily work for others was raised by a number of participants. This suggests that, in the context of child behaviour change interventions, a “one size fits all” approach may not be effective. Interventions of this nature would benefit from recognising this and could potentially offer different strategies depending on the child's and/or mother's circumstances. Traditional and new communication technology delivery modes afford the potential to tailor and personalise the information provided to participants which, in addition to reducing attrition, may help to make parents more receptive to intervention messages [14].

An interesting point raised by some mothers was the need to ensure that any information given to parents is realistic and nonjudgemental. Mothers commented that they would be more receptive to information if it was presented in a way that was understanding to the demands of parenting and different family circumstances. Using a nonjudgemental and nonconfrontational approach (as opposed to a didactic or coercive approach) is recognised as being important in behaviour change interventions [15] and may perhaps be even more pertinent for parents of young children and mothers experiencing disadvantage.

Despite many mothers commenting that they liked to receive information or advice from other parents or their friends or family (i.e., “lay” people), the need for websites to provide credible and reputable content was frequently mentioned. In the context of looking at specific websites, mothers commented that they liked the inclusion of links to other websites as it made the information presented seem more credible. In addition, when asked for recommendations for researchers designing programs, mothers reiterated that the information should be reliable and evidence-based. This suggests that while parents may like to talk with other parents to get ideas and advice, when it comes to information provided to parents in interventions, they may prefer to know that the sources are credible.

A major strength of the current study is that the needs of the target audience (or end users) were investigated. Involvement of key stakeholders or end users has been shown to lead to greater acceptability and engagement with interventions and to improved efficacy [1, 2]. A limitation of the current study is the relatively low response from regional mothers (compared to urban mothers). This may be a result of the recruitment strategies not having as wide a reach in the regional area (due to a smaller overall population), or it may be that mothers in these areas are less interested or able to participate in research. However, data saturation was reached with both the urban and regional mothers; hence, continuing to recruit regional mothers may not have resulted in any additional themes. A further limitation is that, despite recruiting from areas classified as the most disadvantaged, approximately half of the participating mothers were university educated. While this is a lower proportion than that in many studies and overrepresentation of higher-educated women in research studies is commonplace [4, 5], given that education level is a useful proxy for socioeconomic disadvantage, this suggests that the demographic of interest (i.e., socioeconomically disadvantaged mothers) may not have been solely captured. It is important to note that fewer than half of the regional mothers reported that they were living with a partner (compared with almost all of the urban mothers). Single parents as a group are known to experience more social disadvantage [16], suggesting that perhaps the regional mothers may have been more representative of the target population (mothers experiencing disadvantage). Moreover, results from this study are reported by low versus high education level in addition to urban versus regional classification. While some differences were observed in preferences of mothers with low education compared to mothers with high education, responses were mostly similar across education levels, suggesting that new communication technology may benefit mothers across all SEPs. Finally, it is also important to note that the recruitment strategy used may have introduced selection bias. It is possible that mothers who were interested in participating in the study may have had a preceding interest in receiving information online or may have different views to mothers who did not participate.

## 5. Conclusions

In conclusion, urban and regional, high and low educated mothers in this study held favourable views on the potential of receiving information via new communication technologies. Given the nature of the study design, however, it is important to consider that many of the findings from this study are mothers'* anticipated *needs and preferences. That is, when mothers reported their program preferences, they were reporting what they could foresee wanting or needing from a program. In reality, if they are enrolled in a behaviour change program, their views on things such as the number of times per week they would like to receive text messages or emails may change. Therefore, it will be important for future behaviour change programs targeting this population to undertake and report process evaluation to determine what mothers liked or disliked, further informing behaviour change research in this population.

## Figures and Tables

**Table 1:** Urban and regional maternal, child, and household characteristics.

	Total (*n* = 32) *n* (%)	Urban (*n* = 22) *n* (%)	Regional (*n* = 10) *n* (%)
*Maternal characteristics*			
Age, *mean (SD)*	*33.4 (6.1)*	*33.9 (6.4)*	*32.4 (5.7)*
Education level			
Year 10 or equivalent	4 (13)	2 (9)	2 (20)
Year 12/trade/diploma	13 (41)	10 (46)	3 (30)
University degree or higher	15 (47)	10 (46)	5 (50)
Employment status			
Full-time	3 (9)	3 (14)	0 (0)
Part-time	13 (41)	10 (46)	3 (30)
Home duties	10 (31)	5 (23)	5 (50)
Student/unemployed/other	6 (19)	4 (18)	2 (20)
Hours/week in paid employment, *mean (SD)*	*12.6 (12.7)*	*14.5 (13.9)*	*8.3 (8.6)*
Marital status			
Married/de facto/living together	25 (78)	21 (96)^*∗*^	4 (40)^*∗*^
Separated/divorced	4 (13)	0 (0)	4 (40)
Never married	4 (13)	1 (5)	3 (30)
Another child in the home	16 (50)	13 (59)	3 (30)
*Child characteristics*			
Age, *mean (SD)*	*2.5 (0.9)*	*2.6 (0.8)*	*2.4 (1.0)*
Child sex			
Male	14 (44)	11 (50)	3 (30)
Female	18 (56)	11 (50)	7 (70)
Child lives with mother all/most of the time	32 (100)	22 (100)	10 (100)
*Household characteristics*			
Electronics/technology in the home^†^			
Mobile phone with internet	30 (94)	21 (96)	9 (90)
Desktop computer	9 (28)	6 (27)	3 (30)
Laptop computer	26 (81)	17 (77)	9 (90)
Internet access	28 (88)	20 (91)	8 (80)
Tablet	19 (59)	14 (64)	5 (50)
Device used most to access Internet			
Smartphone	14 (44)	10 (46)	4 (40)
Laptop/desktop computer	7 (22)	3 (14)	4 (40)
Tablet	7 (22)	5 (23)	2 (20)
Total weekly hours spent browsing internet for general information,* mean (SD)*	*7.4 (8.8)*	*7.7 (8.2)*	*6.7 (10.2)*

^*∗*^Statistically significant difference between urban and regional groups; ^†^multiple responses were permitted so percentages do not total 100%.

**Table 2:** Urban/regional and low/high educated mothers' access of parenting information.

Question	Total (*n* = 32) *n* (%)	Urban (*n* = 22) *n* (%)	Regional (*n* = 10) *n* (%)	Low educated (*n* = 17) *n* (%)	High educated (*n* = 15) *n* (%)
Where mothers go to access information about parenting and their child^*∗*^					
Internet (Google search, specific websites, Facebook, forums, email newsletters)	27 (84)	20 (91)	7 (70)	13 (76)	14 (93)
Talking with lay people (other parents, family, friends)	19 (59)	16 (73)	3 (30)	9 (53)	10 (67)
Health professionals (doctors, maternal child health nurse, nurse-on-call)	16 (50)	10 (46)	6 (60)	9 (53)	7 (47)
Books/library/magazines	15 (47)	11 (50)	4 (40)	6 (35)	9 (60)
Childcare professionals (childcare staff, playgroup leaders)	4 (13)	1 (5)	3 (30)	2 (12)	2 (13)
Own experience (with older children, other younger family members)	2 (6)	1 (5)	1 (10)	2 (12)	0 (0)
TV, DVDs, radio	2 (6)	1 (5)	1 (10)	1 (6)	1 (7)
How mothers best like to learn about things relating to children/parenting^*∗*^					
Talking with lay people (other parents, family, friends)	24 (75)	12 (55)	2 (20)	9 (53)	5 (33)
Reading (books, magazines, other written materials)	15 (47)	10 (46)	5 (50)	8 (47)	7 (47)
Internet	14 (44)	9 (41)	5 (50)	6 (35)	8 (53)
Health professionals	7 (22)	4 (18)	3 (30)	2 (12)	5 (33)
Watching videos, listening to audio books	5 (16)	3 (14)	2 (20)	2 (12)	2 (13)
Experiential learning	4 (13)	4 (18)	0 (0)	1 (6)	3 (20)
Own upbringing	2 (6)	2 (9)	0 (0)	1 (6)	1 (7)
Practical courses (e.g., sleep school)	1 (3)	0 (0)	1 (10)	1 (6)	0 (0)
Did mothers previously look for information about children's play?					
Yes	21 (66)	14 (64)	7 (70)	7 (41)	14 (93)
No	11 (34)	8 (36)	3 (30)	10 (59)	1 (7)
Did mothers previously look for information about children's screen time?					
Yes	20 (63)	15 (68)	5 (50)	5 (29)	7 (47)
No	12 (38)	7 (32)	5 (50)	12 (71)	8 (53)

^*∗*^Percentages may not add up to 100% as mothers may have had multiple responses.

**Table 3:** Urban/regional and low/high educated mothers' program preferences.

Question	Total (*n* = 32) *n* (%)	Urban (*n* = 22) *n* (%)	Regional (*n* = 10) *n* (%)	Low educated (*n* = 17) *n* (%)	High educated (*n* = 15) *n* (%)
How mothers would prefer to receive information about children's play and screen time if they were involved in a program^*∗*^					
Email	16 (50)	11 (50)	5 (50)	8 (47)	8 (53)
Paper-based	14 (44)	11 (50)	3 (30)	8 (47)	6 (40)
Online (websites, blogs)	8 (25)	4 (18)	4 (40)	3 (18)	5 (33)
Face-to-face (e.g., group session)	7 (22)	6 (27)	1 (10)	3 (18)	2 (13)
Video	2 (6)	2 (9)	1 (10)	1 (6)	1 (7)
Health professionals	1 (3)	0 (0)	1 (10)	0 (0)	1 (7)
How mothers would like to receive text messages with information about children's play					
Yes	25 (78)	18 (82)	7 (70)	13 (76)	12 (80)
No, not long enough	4 (13)	1 (5)	3 (30)	2 (12)	0 (0)
No, would prefer other forms	2 (6)	2 (9)	0 (0)	2 (12)	0 (0)
Maybe but would prefer other forms	1 (3)	1 (5)	0 (0)	0 (0)	1 (7)
How often mothers would like to receive text messages					
Once a week	14 (44)	10 (46)	4 (40)	6 (35)	8 (53)
Once a month	7 (22)	5 (23)	2 (20)	4 (24)	3 (20)
As often as necessary	3 (9)	2 (9)	1 (10)	3 (18)	0 (0)
Daily	3 (9)	2 (9)	1 (10)	1 (6)	2 (13)
Once a fortnight	2 (6)	1 (5)	1 (10)	0 (0)	2 (13)
Twice a week	1 (3)	1 (5)	0 (0)	1 (6)	0 (0)
Every few months	1 (3)	1 (5)	0 (0)	1 (6)	0 (0)
Never	1 (3)	0 (0)	1 (10)	1 (6)	0 (0)
How mothers would like links to reputable websites within text messages					
Yes	29 (91)	20 (91)	9 (90)	15 (88)	14 (93)
No, would prefer to get links via email	2 (6)	2 (9)	0 (0)	1 (6)	1 (7)
No, would not want the text messages at all	1 (3)	0 (0)	1 (10)	1 (6)	0 (0)
How mothers would like a dedicated website with information about children's play					
Yes	29 (91)	20 (91)	9 (90)	15 (88)	14 (93)
No, too many websites already	1 (3)	0 (0)	1 (10)	1 (6)	0 (0)
No, too boring (would need other information as well)	1 (3)	1 (5)	0 (0)	0 (0)	1 (7)
No, would not use it	1 (3)	1 (5)	0 (0)	1 (6)	0 (0)
How often mothers would visit such a website					
Once a week	11 (34)	7 (32)	4 (40)	7 (41)	4 (27)
Daily	6 (19)	6 (22)	0 (0)	4 (24)	2 (13)
Dependent on development/concerns, time, how often it is updated	4 (13)	2 (9)	2 (20)	1 (6)	3 (20)
Once a fortnight	3 (9)	3 (14)	0 (0)	1 (6)	2 (13)
A few times a week	2 (6)	0 (0)	2 (20)	1 (6)	1 (7)
Once a month	2 (6)	1 (5)	1 (10)	1 (6)	1 (7)
Every few months	2 (6)	2 (9)	0 (0)	1 (6)	1 (7)
Every few weeks	1 (3)	1 (5)	0 (0)	0 (0)	1 (7)
Never	1 (3)	0 (0)	1 (10)	1 (6)	0 (0)
How mothers would like receiving email reminders to visit the website					
Yes	28 (88)	19 (86)	9 (90)	15 (88)	13 (80)
No, receive too many emails already, would find it annoying	3 (9)	2 (9)	1 (10)	2 (12)	1 (7)
No, unnecessary (would remember if interested)	1 (3)	1 (5)	0 (0)	0 (0)	1 (7)
How often mothers would like to receive these sorts of emails					
Once a week	12 (38)	7 (32)	4 (40)	7 (41)	4 (27)
Once a fortnight	7 (22)	5 (23)	2 (20)	3 (18)	4 (27)
Once a month	4 (13)	2 (9)	2 (20)	1 (6)	3 (20)
Every few months	3 (9)	3 (14)	0 (0)	1 (6)	2 (13)
Only when something is updated	2 (6)	1 (5)	1 (10)	1 (6)	1 (7)
Daily	1 (3)	1 (5)	0 (0)	1 (6)	0 (0)
As little as possible	1 (3)	1 (5)	0 (0)	1 (6)	0 (0)

^*∗*^Percentages may not add up to 100% as mothers may have had multiple responses.

**Table 4:** Urban/regional and low/high educated mothers' website preferences.

Question	Total (*n* = 32) *n* (%)	Urban (*n* = 22) *n* (%)	Regional (*n* = 10) *n* (%)	Low educated (*n* = 17) *n* (%)	High educated (*n* = 15) *n* (%)
Pictures on websites					
Good	27 (84)	19 (86)	8 (80)	14 (82)	14 (93)
Good, as long as they serve a purpose	5 (16)	3 (14)	2 (20)	3 (18)	1 (7)
Informative videos on websites					
Good	22 (69)	16 (73)	6 (60)	10 (59)	12 (80)
Do not like/use them	5 (16)	4 (18)	1 (10)	4 (24)	1 (7)
Good, depending on the topic	4 (135)	2 (9)	2 (20)	2 (12)	2 (13)
Good, but uses a lot of data	1 (3)	0 (0)	1 (10)	1 (6)	0 (0)
Written information on websites					
Good	17 (53)	13 (59)	4 (40)	11 (65)	6 (40)
Good, but not too much (i.e., needs to be in dot points, small paragraphs)	8 (25)	5 (23)	3 (30)	3 (18)	5 (33)
Good, but should be accompanied with pictures and/or videos	6 (19)	3 (14)	3 (30)	2 (12)	4 (27)
Good, but needs to be in layman's terms	1 (3)	1 (5)	0 (0)	1 (6)	0 (0)
